# Correlation of Bacterial Infections in Women With and Without Intrauterine Devices

**DOI:** 10.7759/cureus.86814

**Published:** 2025-06-26

**Authors:** Alexander Golden, Daphne E Sanchez, Colette Cipriano, Rebecca L Sanchez, Rebecca Cipriano

**Affiliations:** 1 Obstetrics and Gynecology, Baystate Medical Center, Springfield, USA; 2 Osteopathic Medicine, University of the Incarnate Word School of Osteopathic Medicine, San Antonio, USA; 3 Biology, Villanova University, Villanova, USA; 4 Applied Biomedical Sciences, University of the Incarnate Word School of Osteopathic Medicine, San Antonio, USA; 5 Obstetrics and Gynecology, Healthy Woman OBGYN, Freehold, USA

**Keywords:** bacterial vaginosis, intrauterine device, iud, mycoplasma, naat, ureaplasma

## Abstract

Introduction: The goal of this study is to evaluate the correlation of* *bacterial infections acquired through sexual transmission, including *Neisseria gonorrhea (NG)*, *Chlamydia trachomatis (CT)*, *Mycoplasma hominis (Myc)*, *Ureaplasma urealyticus (Urea),* and bacterial vaginosis (BV) in women and those who seek gynecologic care with a history of intrauterine device (IUD) placement versus those without a history of IUD placement.

Methods: A deidentified, retrospective analysis, with 234 test results within a large obstetrics and gynecology (OBGYN) practice in both Monmouth and Ocean County, New Jersey, from 2019 to 2020, was used to determine positivity rates of* *bacterial infections in both IUD users and non-IUD users. Fisher's exact and chi-squared tests were used to determine any association between the two groups.

Results: The study determined statistical variation among the two groups for BV positivity (p < 0.001), CT/NG (p = 0.03), and concurrent infections (p = 0.002). However, no statistical difference was noted for Myc/Urea infections (p = 0.05, 95% CI: 0.89-20.88, OR: 3.60). Furthermore, no statistical variation was determined by age. However, there was a statistical difference in concurrent infections by IUD types (p = 0.02, 95% CI: 0.00-0.87, OR: 0).

Conclusion: This study supports a statistically significant association between gonorrhea, chlamydia, and BV in women and those who seek gynecologic care with IUD usage. Our findings also reveal that age and nucleic acid amplification test (NAAT)-confirmed positivity for *Mycoplasma*/*Ureaplasma *do not exhibit statistically significant correlations between IUD users and non-IUD users. Given the clinical significance of providing patient education towards appropriate contraception use, our study furthers existing literature by shedding light on the correlation of IUD usage with infection.

## Introduction

Vaginitis is one of the most common reasons a patient visits their obstetrician and gynecologist (OBGYN) at an estimated 10 million annual office visits [1]. Although often treated successfully, cases of recurrent bacterial vaginitis (BV) can be frustrating for both patients and practitioners. On a foundational level, women and those who seek gynecologic care will experience clinical symptoms of BV when the protective *Lactobacilli *spp. decrease in number, regardless of etiology, allowing for the excess production of local anaerobic bacterial species [2]. Diagnosing BV requires the use of Amsel’s criteria or Nugent’s score [2]. Amsel’s criteria contain four conditions, three of which are needed for a positive diagnosis: (1) homogenous vaginal discharge, (2) a positive whiff test, (3) presence of clue cells, and/or (4) a vaginal pH > 4.5 [3]. Nugent’s score varies from 0 to 10, where a score of 7-10 is considered a positive diagnosis for BV [3]. There are also many readily available vaginal cultures to help guide treatment used every day in clinical practice, including FDA-approved swabs such as the BD Max vaginal panel and laboratory-developed tests such as NuSwab VG [4].

As complex as BV is to both identify and treat, sexually transmitted infections (STIs), such as *Mycoplasma hominis* and *Ureaplasma* *urealyticum*, can form a biofilm within the vagina and mimic BV symptoms, which can be difficult to treat [5]. Thus, an inherent risk of meddling with this bacterial environment is likely with the introduction of a foreign device into the vaginal cavity [6]. Once the biofilm has formed upon a surface within the vagina, there is an increase in sialidase, prolidase, acetate succinate, and vaginolysin. Detailed information regarding these virulence factors can be found in a study [7]. Subsequently, protective mucosal antibodies, the production of IL-8, neutrophils, and mucin decrease, which ultimately inhibits both the adaptive and innate immune responses [5]. Persistent infections have been recorded as this biofilm contributes to evasion of the host immune system as well as antibiotic resistance [5]. It is reported that over 50% of patients who are treated with BV will experience a recurrence within 6-12 months [5].

Since 1995, the United States (US) has exhibited an increase in long-acting reversible contraception in the form of intrauterine devices (IUDs) [8]. With failure rates equivalent to 0.08% in the copper-containing IUD and 0.02% in the levonorgestrel-containing IUD, more and more patients seek this form of contraception because of its efficacious history and/or its minimal burden of responsibility to the user, both financially and regarding follow-up care [8]. Per the United Nations’ World Family Planning Report of 2022, 16.8% of patients of reproductive age (15-49 years old) worldwide use an IUD as their contraceptive method [9]. The types and how many years the device is in place are many, and the choice of IUD can depend on bleeding profile, parity, and other factors [8]. Placed across the endometrial cavity after identifying the cervix and the uterus, the IUD can be left alone, barring any complications, for anywhere from 3 to 10 years based on the device agreed upon by the provider and the patient [8]. Some of the highest yield complications after placement are a shifting of the device from its desired location, accidental removal, or patient dissatisfaction, either with mood or infection [8]. For some patients, the benefits of adequate contraception and/or controlling the effects of menorrhagia do seem to outweigh the risks of IUD therapy [8].

Objective

Given the interrelationship between the vaginal microbiota and BV, this study primarily aimed to compare the prevalence of BV and STIs between IUD users and non-users. As a secondary objective, it evaluated whether the type of IUD (hormonal vs. non-hormonal) modified this risk. Our hypothesis is that the number of infections in patients will increase because of the IUD serving as a nidus for pathogenic bacteria to flourish.

## Materials and methods

Samples were taken from a large OBGYN practice with offices in Monmouth and Ocean County, New Jersey, from 2019 to 2020. During this timeframe, 234 entries from that pool of data were retroactively deidentified by a provider on-site before being shared for analysis. Each deidentified entry contained information on results of nucleic acid amplification tests (NAAT) for *Mycoplasma *and *Ureaplasma *(Myc/Urea); BV, including yeast, as defined by the standard vaginitis panel (see Aptima Multitest Swab below); chlamydia/gonorrhea (CT/NG); IUD placement; IUD status (i.e., removed, in place, replaced, etc.); and IUD type (Mirena, Kyleena, or ParaGard). Entries were combined into one category of “positive” if NAAT revealed *Mycoplasma *or *Ureaplasma *and if CT/NG revealed growth of either organism. Concurrent infections were determined by entries that tested positive for at least one positive NAAT result, a BV/yeast result, and one positive CT/NG. IUD types were also categorized based on their hormonal status, with Mirena and Kyleena being grouped together (i.e., hormonal) and ParaGard being evaluated independently (i.e., non-hormonal). Age was categorized in five-year intervals (i.e., 15-19, 20-24, 25-29, etc.). Table 1 presents detailed information regarding these virulence factors [7].

**Table 1:** Definition of the top virulence factors that contribute to bacterial vaginosis. Source: Ref [7]

Virulence Factors	
Sialidase	Cleaves the terminal sialic acid residues of carbohydrate polymers found in the vaginal microbiome; contributes to adhesion and biofilm formation
Prolidase	Proteolytic enzyme that degrades the extracellular matrix (esp. mucin); modulates the immune response
Vaginolysin	Cholesterol-dependent, pore-forming protein with an affinity for red blood cells

Data were analyzed using R (version 4.3.2; R Development Core Team, Vienna, Austria), an open-source programming software language for statistical analysis, and G power software (version 3.1.9.7; The G*Power Team, Germany). G-Power was used to calculate the minimum sample size (n = 78) for a power of 0.8 and a significance value of p < 0.05. Fisher's exact test was used to determine the difference between the use of IUD and the rates of Myc/Urea infections, CT/NG infections, and concurrent infections. A chi-squared test was used to determine the difference between the use of IUDs and the rates of BV/yeast infections. Among IUD users, a subsequent Fisher's exact test was used to determine the relationship between CT/NG infections and concurrent infections by IUD type (i.e., non-hormonal, hormonal). A subsequent chi-squared test was used to determine BV infections by IUD type. A logistic regression model was also used to adjust for age by BV, CT/NG, and concurrent infections among IUD users.

IRB approval was received for de-identified retrospective analysis as the project was deemed as non-regulated research (NRR).

Laboratory methods

The vaginitis panel nucleic acid amplification test (NAAT) is a molecular laboratory test that is commonly known as the Aptima Multitest Swab in the field of OBGYN. It was copyrighted by Hologic. The swab is removed from its package by a practitioner and placed about 5 cm into the vagina, past the introitus, and rotated around all surfaces of the patient’s vaginal walls for about 10-30 seconds. The swab is then placed in its transport tube, capped, and sent for molecular testing. The transport media within the transport tube is a guanidine-free, non-toxic solution that helps preserve the integrity of the sample so that it can be accurately processed by the laboratory facility. The media will lyse the cells, which will subsequently release the RNA into the solution. Once the sample is run by the facility, highly conserved regions of the RNA can be hybridized to a target if one or more of the vaginitis infections are present [10]. Through magnetized molecular methodology, excess regions can be washed away, and transcription-based amplification of target sequences can be performed. Particularly, Moloney murine leukemia virus reverse transcriptase and T7 RNA polymerase facilitate this process to ultimately produce a DNA template per the central dogma of molecular biology. Detection of a vaginal microbe can then be generated once single-stranded nucleic acid torches hybridize to a target segment, if present, which ultimately allows a fluorescent signal to be emitted when excited by a light source. Ultimately, the Aptima can detect up to seven infections. Results are reported as positive or negative. Sensitivity is greater than or equal to 95% [10].

This test’s data can be limited by the following: practitioner error, practitioner contamination, general contamination of the sample by the lab personnel, patient’s menarcheal status, presence or absence of symptoms, duration of symptoms, previously treated infections with prescription medications, presence or absence of over-the-counter medications used by the patient, and use of intravaginal devices (tampons, dilators, etc.). Swabs can also be obtained by the clinician or patient (self-swab); thus, self-swabbing can also affect results. Incomplete records were excluded from our study as well as any results that were indeterminate.

## Results

During this period, 234 tests were extracted, with 116 tests coming from IUD users and 118 coming from non-IUD users. Of the 234 tests, a total of 13 (5.56%) positive NAATs for *Mycoplasma*/*Ureaplasma* (Table 2) were observed, with 10 being from IUD users and three from non-IUD users. No statistical variation was determined among the two groups (p = 0.05, 95% CI: 0.89-20.89, OR: 3.60). A significant difference was determined between IUD use and BV positivity with 39 positive results being from IUD users and 10 being from non-IUD users (p < 0.001, df = 1, Φ = 0.31 ). Similarly, a statistical variation was determined for the rates of CT/NG infections, with three positive results being from non-IUD users and 11 being from IUD users (p = 0.03, 95% CI: 1.02-22.9, OR: 3.99). Furthermore, there was a statistical difference among concurrent infections between the two groups (p = 0.002, 95% CI: 1.75-73.17, OR: 7.90).

**Table 2:** Characteristics of IUD and non-IUD users by age group, IUD type, and bacterial infections (Fisher's exact test and chi-squared test). Myc/urea = *Mycoplasma *or urea infections. CT/NG = Chlamydia or Gonorrhea. BV = Bacterial vaginosis Fisher's exact test was used for Myc/Urea, CT/NG, and concurrent infection. A chi-squared test was used for BV infections.

IUD Use	Yes	No	P-value
Age Group			
< 19	0 (0.0)	6 (5.1)	
20-24	16 (13.8)	33 (28.0)	
25-29	26 (22.4)	18 (15.3)	
30-34	30 (25.9)	13 (11.0)	
35-39	18 (15.5)	11 (9.3)	
40-44	9 (7.8)	18 (15.3)	
45-49	9 (7.8)	19 (16.1)	
50+	8 (6.9)	0 (0.0)	
IUD Type			
Hormonal	88 (75.9)	0 (0.0)	
Non-hormonal	28 (24.1)	0 (0.0)	
No IUD	0 (0.0)	118 (100.0)	
Myc/Urea Infections			0.05
Positive	10 (8.6)	3 (2.5)	
Negative	106 (91.4)	115 (97.5)	
CT/NG Infections			0.03
Positive	11 (9.5)	3 (2.5)	
Negative	105 (90.5)	115 (97.5)	
BV Positivity			<0.001
Yes	39 (33.6)	10 (8.5)	
No	77 (66.4)	108 (91.5)	
Concurrent Bacterial Infection			0.002
Yes	14 (12.1)	2 (1.7)	
No	102 (87.9)	116 (98.3)	

The average age among patients was 32.7 years (SD = 9.1), with the range being 17-55 years old. A logistic regression model was performed to adjust for age among these infections. No statistical variation was determined for CT/NG infections, BV infections, and concurrent infections by age (Table 3).

**Table 3:** Logistic regression model of age of BV, CT/NG, and concurrent infections among IUD users. n = 116, missing numbers = 0, BV = Bacterial vaginosis, CT/NG = Chlamydia or gonorrhea

Variables		BV Infections																CT/NG Infections							Concurrent Infections				
Predictor		OR						95% CI						P-value				OR			95% CI		P-value		OR		95% CI	P-value	
Age		1.02						0.97-1.07						0.388				0.99			0.91-1.06		0.706		1.00		0.94-1.07	0.908	
AIC								151.4													76.6						89.4		
C-Statistic								0.545													0.546						0.516		
Chi-sq(8)						8.40 (p=0.395)														4.64 (p=0.796)						9.64 (p=0.291)			

IUD users were also broken down by IUD type, with 88 (75.9%) patients using a hormonal-based IUD, such as Kyleena and Mirena, and 28 (24.1%) using a non-hormonal IUD, such as ParaGard (Table 4). No statistical difference was observed for CT/NG (p = 0.30) and BV infections (p = 0.38, df = 1, Φ = -0.10) among these two groups. However, a statistical difference for the rates of concurrent infection among these IUD types was observed, with 0 positive concurrent infection results within the non-hormonal group and 14 positive results within the hormonal group (p-value = 0.02, 95% CI: 0.00-0.87, OR: 0.00).

**Table 4:** Rates of BV, CT/NG, and concurrent infections among IUD users by IUD type (Fisher's exact test and chi-squared test). BV = Bacterial vaginosis. CT/NG = Chlamydia or gonorrhea ^ = Chi-squared test performed. * = statistically significant (p-value < 0.05)

Variables	Hormonal	Non-Hormonal	P-value
BV Infections			0.38^
Positive	32 (82.1)	7 (17.9)	
Negative	56 (72.7)	21 (27.3)	
CT/NG Infections			0.30
Positive	10 (90.9)	1 (9.1)	
Negative	78 (74.3)	27 (25.7)	
Concurrent Infections			0.02*
Positive	14 (100.0)	0 (0.0)	
Negative	74 (72.5)	28 (27.5)	

## Discussion

Our data show that there was a statistically significant difference in BV, STI, and concurrent infections among women and those who seek gynecologic care who have an IUD. Thus, our study suggests IUDs may serve as a nidus for pathogens, as we hypothesized. While data from previously performed studies investigating this topic within the last five years in the US are sparse, we hope that our results can serve as a stepping-stone for further investigation.

Comparison of our study to those previously performed shows that data regarding this topic are controversial and demonstrate conflicting results. An integrative review of cross-sectional studies published in 2023 by Daniel et al. showed that all IUD users combined may have an increased point prevalence of BV compared with non-IUD users [11]. However, these studies did not delineate which type of IUD was used [11]. A prospective cohort study performed in 2021 showed that women who use the copper IUD experienced a 1.28-fold risk for vaginal bacterial infections [12]. In a study done in 2012 by Madden et al., their team was able to find that the crude analysis of their data revealed significance for BV, but ultimately showed no significant association after factoring in confounding variables [13]. Similarly, a study performed by Meirik in 2007 demonstrated that there was no statistical significance between IUD users and BV [14]. Even though Madden et al. did not find significant associations after adjusting for cofounders, the study was able to determine an association between intermediate vaginal flora and BV in IUD users [13]; thus, it is likely that the changes in pathogen prevalence over the past decade may have played a role in determining our association between BV and IUD use.

A strength of our study is that we are working with patients in the United States of America within the last 5-10 years.. Moreover, there are clear outcome measures, inclusion of demographic information, and inclusion of IUD types involved in the subject pool. Limitations to our study include that the subjects were from one practice in one location of the US, which may err on the side of selection bias. In addition, the sample size is rather small. Thus, the ability to generalize our results to the rest of the population may not be valid. We also did not report history of previous or repetitive IUD use, first-time users, and duration of IUD placement, thus contributing to temporal ambiguity and limiting our study's ability of establishing causality between vaginal infections and IUD use. Furthermore, our study did not record information regarding exogenous hormone use concomitantly, patient’s sexual activity, multiple sexual partners, condom use, prior STI history, or other comorbidities (e.g., diabetes, autoimmune diseases, etc.) the subject may have had, which limited the study's ability to control for cofounding variables.

Future studies should certainly include a larger multi-center collaboration from different regions of the US. We also recommend a future study that explores infectivity in those using hormonal IUDs versus the copper-containing IUD in today’s market. We suspect that looking into comorbidities, especially autoimmune diseases and those involving the gastrointestinal tract, may also alter the rate of positivity for those who have an IUD placed. Because sexual intercourse increases the risk of contracting BV, it would be worth exploring if patients with an IUD were using condoms for prevention of infection and/or if they had multiple sex partners [15]. Furthermore, because of the preliminary nature of our study, it is imperative that future longitudinal studies be conducted to establish causality between BV and IUD use. That is, does the IUD cause BV, or are women with BV more likely to use an IUD? In addition, future studies may focus on detailed analysis of the vaginal microbiome before and after IUD insertion.

Given the scarcity of recent data on this topic in the US, our results provide a baseline for future investigations to delve deeper into the intricate associations between IUD usage and bacterial infections. The major clinical significance of this study is that it can help guide physicians in counseling their patients, especially those of an early reproductive age group (under 35), those with histories of recurrent vaginitis, and autoimmune or gastrointestinal disease histories about alternative options for contraception. Additionally, it can be considered a risk when counseling about the risks and benefits of IUD placement. The insights gained from our study may pave the way for more comprehensive research, fostering a better understanding of the dynamics involved and ultimately influencing clinical approaches and counseling practices in women and those who seek gynecologic care's reproductive health.

## Conclusions

In conclusion, our study provides preliminary information regarding the intricate association between IUD usage and bacterial infections. Our study presents evidence pointing towards a statistically significant difference in bacterial vaginosis infections among women with IUDs. Our findings also reveal that age and IUD type do not exhibit statistically significant correlations among IUD users, but further studies could explore more. Widespread testing of *Mycoplasma*/*Ureaplasma *is not performed regularly and sometimes dismissed as routine discharge. The controversies observed in comparing our study with existing literature underline the complexity of this subject. As the use of IUDs continues to rise in the US, understanding the risks and benefits of their use needs to be further explored. Our study aims to provide a baseline for this topic. Over time, findings may prompt changes in counseling practices, which can benefit the patient enormously by steering patients with a history of infections or multiple sex partners toward another modality of birth control. Overall, this study adds evidence that research in women’s health is needed. We hope that our findings will serve as a stepping stone for more longitudinal studies in assisting women to make the right decisions in their reproductive health.
